# Beta-amyloid production in neurons is regulated by astrocyte-derived cholesterol

**DOI:** 10.1038/s42003-021-02644-7

**Published:** 2021-09-22

**Authors:** Karli Montague-Cardoso

**Affiliations:** Communications Biology, https://www.nature.com/commsbio

## Abstract

The accumulation of amyloid β (Aβ) in the brain is an established feature of Alzheimer’s disease, however mechanisms that regulate Aβ accumulation are not fully understood. In a recent study, Wang et al show that Aβ accumulation in neurons is tightly regulated by cholesterol production in astrocytes. This finding paves the way for future work that will establish whether the selective removal of Aβ by targeting this mechanism has therapeutic potential.

Alzheimer’s Disease (AD) pathology is characterised by inflammation, tau tangles and the accumulation of amyloid β (Aβ). The most common genetic risk factor for sporadic AD is variation in a cholesterol transport protein, apolipoprotein E (apoE) and it has been previously shown in cell culture that apoE is linked to the production of Aβ. However, whether apoE regulation of Aβ also occurs in vivo and could constitute a potential therapeutic target has remained unclear.

In a recent study Wang et al.^1^ used superresolution imaging in mouse brains to show that apoE uses astrocyte-derived cholesterol to transport neuronal amyloid precursor protein (APP) across neuronal cell membranes by regulating its passage in and out of lipid compartments, known as lipid rafts. When inside the lipid rafts, APP interacts with the enzymes β- and γ-secretase to form Aβ. Wang et al went on to show that by deleting astrocyte cholesterol synthesis specifically, both amyloid and tau burden were significantly decreased in a mouse model of AD. In terms of the mechanism underlying this protective effect, they showed in primary neuronal cell culture that either treatment with cholesterol-free apoE or genetically knocking down cholesterol synthesis in astrocytes resulted in APP being transported out of the lipid rafts. Being outside of the rafts exposed APP to a different secretase enzyme - α-secretase - which resulted in the production of soluble APP-α. Unlike Aβ, APP-α exerts protective effects over neurons. Importantly, changes in cellular cholesterol had no effects on the trafficking of α-, β-, and γ-secretase. This strongly implies that the ratio of Aβ to sAPP-α in neurons is regulated by trafficking of the substrate rather than trafficking of the enzymes.

Taken together, Wang et al. conclude that the availability of cholesterol in astrocytes regulates Aβ production in neurons by affecting the trafficking of its substrate. This advances our understanding of the underlying pathomechanisms of AD and could, at least in part, account for the role of cholesterol-associated genes as a risk factor for AD as well as provide a potential avenue for the development of innovative therapies.

## References

[1] Wang H, Kulas JA, Wang C, Holtzman DM, Ferris HA, Hansen SB (2021). Regulation of beta-amyloid production in neurons by astrocyte-derived cholesterol. Proc Natl Acad Sci USA.

